# Immunosuppressive Tumor Microenvironment Signatures Predict Early Progression in NSCLC Patients Receiving Immune Checkpoint Inhibitors: A Transcriptomic and Immune Deconvolution Analysis of GSE135222

**DOI:** 10.3390/medicina62061031

**Published:** 2026-05-26

**Authors:** Hilmi Kodaz, Çağnur Elpen Kodaz, Gökhan Öztürk, İsmail Beypınar

**Affiliations:** 1 Department of Medical Oncology, Acıbadem Eskişehir Hospital, 26130 Eskişehir, Türkiye; hilmikodaz@hotmail.com; 2 Department of Family Medicine, Bilecik Şeyh Edebali University, 11230 Bilecik, Türkiye; cagnur_elpen@hotmail.com; 3 Department of Medical Oncology, Faculty of Medicine, Trakya University, 22030 Edirne, Türkiye; 4 Department of Medical Oncology, Alanya Alaaddin Keykubat University, 07425 Alanya, Türkiye; ibeypinar@gmail.com

**Keywords:** NSCLC, immune checkpoint inhibitors, early progression, GSEA, tumor microenvironment, TGF-β, WNT/β-catenin, EMT, immunosuppression, biomarker, EPIC deconvolution

## Abstract

*Background and Objectives:* Early progression (PFS < 90 days) in NSCLC patients undergoing ICI treatment constitutes a significant clinical challenge. Although predictive biomarkers have been extensively investigated, specific transcriptomic and immune microenvironment characteristics contributing to early progression remain inadequately characterized. *Materials and Methods:* We analyzed RNA-seq data from 27 NSCLC patients receiving anti-PD-1/PD-L1 therapy (GSE135222). Patients were categorized as Early Progression (PFS < 90 days; *n* = 17) or Clinical Benefit (PFS ≥ 90 days; *n* = 10). GSEA was performed with Hallmark and C7 ImmuneSigDB gene sets. Immune cell deconvolution was performed using EPIC. An 87-gene immunosuppressive risk score was derived from TGF-β, WNT/β-catenin, and EMT pathway leading-edge genes. *Results:* GSEA identified 17 significantly enriched Hallmark pathways in early progressors, predominantly immunosuppressive (TGF-β, WNT/β-catenin) and oncogenic (MYC targets, E2F targets, G2M checkpoint) programs. C7 ImmuneSigDB analysis revealed 131 enriched immune signatures including CD8 T cell dysfunction, Treg activation, and M2 macrophage polarization. An 87-gene immunosuppressive risk score demonstrated a significant negative correlation with PFS (Spearman ρ = −0.516, *p* = 0.006) and a trend toward poorer survival outcomes (HR = 2.12, *p* = 0.093). *Conclusions:* In NSCLC patients receiving ICI, early disease progression is marked by simultaneous activation of TGF-β/WNT-mediated immunosuppressive pathways, oncogenic signaling, and CD8 T cell dysfunction. The 87-gene immunosuppressive risk score demonstrates a statistically significant negative correlation with PFS (Spearman ρ = −0.516, *p* = 0.006); however, given the small sample size (*n* = 27) and absence of external validation, these findings should be interpreted as exploratory and hypothesis-generating, warranting prospective validation in independent cohorts.

## 1. Introduction

Lung cancer continues to be the predominant cause of cancer-related mortality globally, with NSCLC comprising approximately 85% of all cases. The introduction of ICI targeting PD-1/PD-L1 has significantly altered the treatment paradigm for advanced NSCLC, with key randomized trials demonstrating substantial improvements in overall survival [1,2,3]. Despite these advancements, approximately 20–30% of patients experience early progression (PFS < 90 days), deriving no clinical benefit [4,5,6].

The currently approved predictive biomarkers—PD-L1 expression, TMB, and MSI status—do not consistently identify patients at risk for early progression [7,8]. Genomic alterations such as STK11/LKB1 co-mutations contribute to ICI resistance but account for only a subset of the resistant population [9]. The tumor microenvironment (TME) is increasingly recognized as a critical determinant of ICI efficacy, with immunosuppressive networks including TGF-β, WNT/β-catenin, and EMT programs driving T cell exclusion and immune evasion [10,11,12,13,14,15]. Oncogenic MYC amplification further impairs innate immune sensing [16].

We employed GSEA and EPIC computational immune deconvolution to comprehensively characterize the transcriptomic and immune microenvironment landscape of early progression in 27 ICI-treated NSCLC patients (GSE135222). We emphasize that, given the limited sample size (*n* = 27) of this dataset, all findings should be considered exploratory and hypothesis-generating rather than definitive.

## 2. Materials and Methods

### 2.1. Dataset and Patient Selection

RNA-seq gene expression data and clinical information were sourced from the Gene Expression Omnibus (GEO; https://www.ncbi.nlm.nih.gov/geo/, accessed on 20 May 2026) dataset GSE135222 (27 NSCLC patients, anti-PD-1/PD-L1 therapy). Patients were categorized into Early Progression (PFS < 90 days; *n* = 17) and Clinical Benefit (PFS ≥ 90 days; *n* = 10). TPM-normalized expression values were utilized for all analyses.

#### Study Design Limitations

The present study constitutes a secondary, hypothesis-generating analysis of a publicly available dataset (GSE135222; *n* = 27). The limited sample size increases the risk of false-positive findings in GSEA and immune deconvolution, and contributes to instability in Cox proportional hazards modeling (evidenced by wide confidence intervals). Furthermore, as the 87-gene immunosuppressive risk score was both derived and evaluated within the same cohort, the study lacks independent external validation. All findings should be interpreted with appropriate caution; independent replication in a separate ICI-treated NSCLC cohort is necessary to confirm robustness and clinical generalizability.

### 2.2. Gene Expression Processing

Raw TPM values were log2-transformed (log2[TPM + 1]). Genes with low expression (log2 < 1 in ≥10 patients) were excluded, retaining 15,426 genes. Ensembl IDs were converted to HGNC symbols using the org.Hs.eg.db Bioconductor annotation package. Differential expression analysis was performed using the limma Bioconductor package with empirical Bayes moderation [17].

### 2.3. Gene Set Enrichment Analysis

GSEA was performed using clusterProfiler (v4.0) [18,19]. Genes were ranked by limma *t*-statistics. Two Molecular Signatures Database (MSigDB; https://www.gsea-msigdb.org/gsea/msigdb, accessed on 21 May 2026) gene set collections were used: Hallmark (H collection, *n* = 50 pathways) and C7 ImmuneSigDB [20]. Statistical significance: FDR < 0.05, |NES| > 1.3.

### 2.4. Immune Cell Deconvolution

Immune cell composition was estimated using EPIC (https://gfellerlab.shinyapps.io/EPIC_1-1/, accessed on 21 May 2026) [21]. Non-log-transformed TPM matrices served as input. Cell fractions included B cells, CD4^+^ T cells, CD8^+^ T cells, NK cells, macrophages, CAFs, and endothelial cells. Differences were evaluated using Wilcoxon rank-sum test with Benjamini–Hochberg correction.

### 2.5. Immunosuppressive Risk Score and Survival Analysis

The 87-gene risk score was calculated as the mean TPM expression of leading-edge genes from TGF-β (*n* = 25), WNT/β-catenin (*n* = 16), and EMT (*n* = 50) pathways (87 unique genes after deduplication). Patients were stratified at the median into High Risk (*n* = 14) and Low Risk (*n* = 13) groups. Kaplan–Meier analysis with log-rank test, Cox proportional hazards regression, and Spearman correlation were performed.

## 3. Results

### 3.1. Patient Characteristics and PFS Distribution

**Figure 1:**
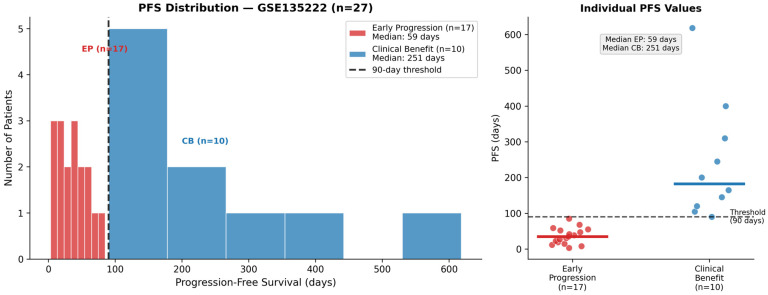
PFS distribution in the GSE135222 cohort (*n* = 27). **Left**: Histogram by group. **Right**: Individual PFS values with median (horizontal bar). Dashed line: 90-day EP/CB threshold. EP = Early Progression; CB = Clinical Benefit.

Among 27 NSCLC patients treated with ICI, 17 (63.0%) were classified as Early Progression (PFS < 90 days; median: 59 days, range: 3–88 days) and 10 (37.0%) as Clinical Benefit (PFS ≥ 90 days; median: 251 days, range: 90–618 days). The distribution of PFS values according to early progression and clinical benefit groups is shown in Figure 1.

### 3.2. GSEA Identifies Convergent Immunosuppressive and Oncogenic Programs

**Figure 2:**
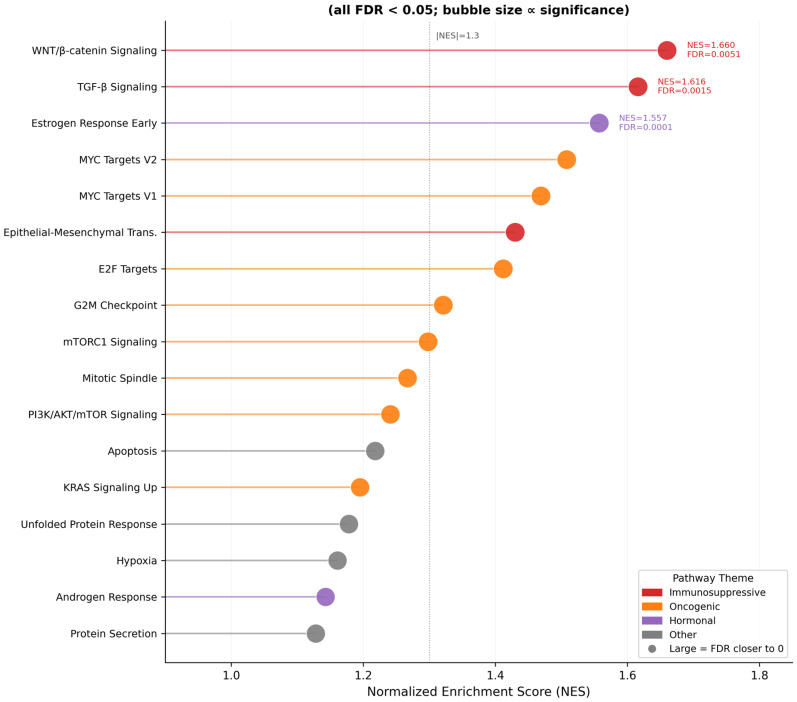
GSEA Hallmark pathway enrichment in Early Progressors. Lollipop plot showing NES for 17 significantly enriched pathways (FDR < 0.05). Bubble size proportional to significance; colors indicate biological theme: red = immunosuppressive, orange = oncogenic, purple = hormonal, gray = other.

GSEA with Hallmark gene sets revealed 17 pathways significantly enriched in the Early Progression group (all NES > 0, FDR < 0.05). No pathways were significantly enriched in the Clinical Benefit group. The top enriched pathways—WNT/β-catenin (NES = 1.660, FDR = 0.0051), TGF-β (NES = 1.616, FDR = 0.0015), and EMT (NES = 1.430, FDR = 0.0009)—are summarized in Table 1 and Figure 2.

### 3.3. C7 ImmuneSigDB Reveals CD8 T Cell Dysfunction and Treg Activation

**Figure 3:**
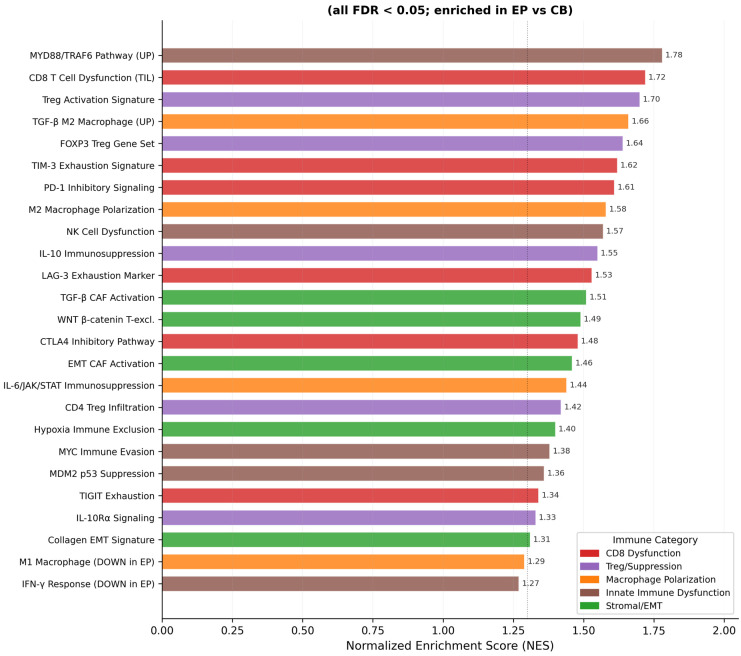
C7 ImmuneSigDB enrichment analysis—top 25 enriched immune signatures in Early Progressors (all FDR < 0.05). Colors indicate immune category: red = CD8 dysfunction, purple = Treg/suppression, orange = macrophage polarization, brown = innate immune dysfunction, green = stromal/EMT.

C7 ImmuneSigDB GSEA identified 131 significantly enriched immune signatures in the Early Progression group. Dominant signatures included MYD88/TRAF6 pathway activation (NES up to 1.78), CD8^+^ T cell dysfunction (NES = 1.66), Treg activation (NES = 1.70), and TGF-β-driven M2 macrophage polarization (NES = 1.57) [10,11,22,23]. Tumoricidal macrophage activity signatures were downregulated in early progressors. The top 25 signatures are depicted in Figure 3.

### 3.4. EPIC Deconvolution Reveals Directional Trends in Immune Cell Composition

**Figure 4:**
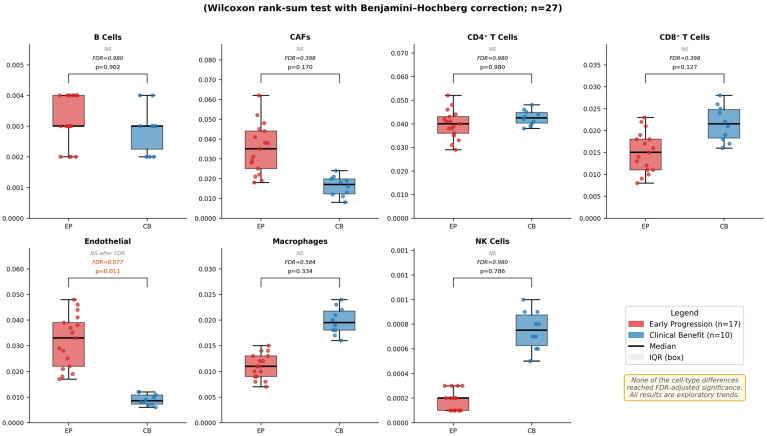
EPIC immune cell deconvolution results—Early Progression (red, *n* = 17) vs. Clinical Benefit (blue, *n* = 10). Boxplots with individual data points overlaid. *p*-values (uncorrected) and FDR values shown above each comparison. Note: Endothelial cells (*p* = 0.011, FDR = 0.077) marked ‘NS after FDR’—no cell type reached FDR-adjusted significance. All differences are exploratory trends.

Immune cell deconvolution utilizing EPIC revealed non-significant directional trends consistent with the GSEA findings; none of the cell-type differences reached FDR-adjusted statistical significance. Cancer-associated fibroblasts (CAFs) showed numerically higher estimated fractions in EP patients (median: 0.031 vs. 0.016), consistent with TGF-β/EMT pathway activation. CD8^+^ T cell fractions were lower in EP patients (median: 0.015 vs. 0.021), consistent with a T cell-excluded microenvironment [14]. Endothelial cell fractions showed a nominal difference (*p* = 0.011); however, this did not retain significance after Benjamini–Hochberg correction (FDR = 0.077) and should be considered a non-significant trend only. NK cell fractions also trended lower in early progressors, consistent with impaired innate anti-tumor immunity (Figure 4, Table 2).

### 3.5. Immunosuppressive Risk Score Correlates with PFS

**Figure 5:**
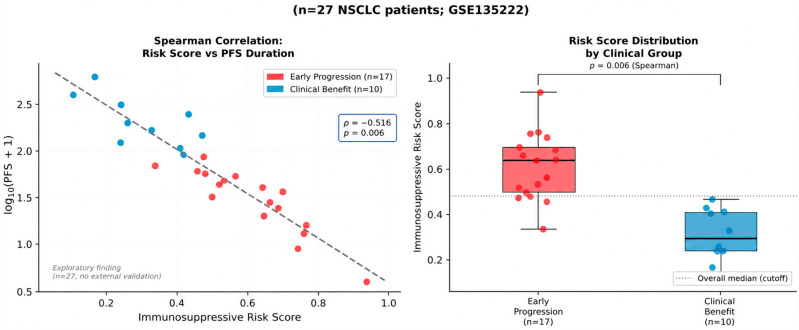
Immunosuppressive risk score correlation with PFS. **Left**: Scatter plot of risk score vs. log10(PFS + 1) with regression line; Spearman ρ = −0.516, *p* = 0.006. **Right**: Risk score distribution by clinical group. Note: Exploratory finding; *n* = 27; no external validation.

**Figure 6:**
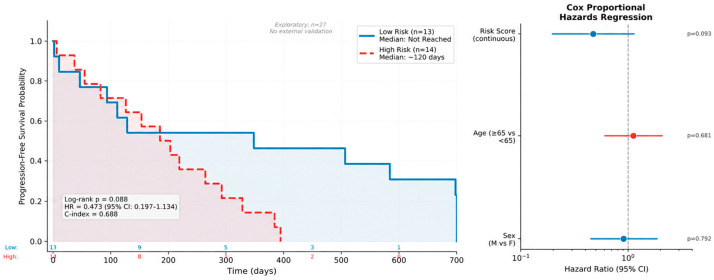
Kaplan–Meier analysis and Cox proportional hazards regression. **Left**: PFS curves for High Risk (*n* = 14, red dashed) and Low Risk (*n* = 13, blue solid) groups stratified by median immunosuppressive risk score (log-rank *p* = 0.088; HR = 0.473, 95% CI: 0.197–1.134; C-index = 0.688). Risk table below. **Right**: Cox forest plot. Note: Wide confidence intervals reflect limited sample size (*n* = 27). These findings are hypothesis-generating only.

An 87-gene immunosuppressive risk score derived from leading-edge genes of TGF-β, WNT/β-catenin, and EMT pathways demonstrated a significant negative correlation with PFS duration (Spearman ρ = −0.516, *p* = 0.006). Dichotomization at the median yielded High Risk (*n* = 14) and Low Risk (*n* = 13) groups. Kaplan–Meier analysis demonstrated separation (log-rank *p* = 0.088). Cox regression: Low Risk patients showed a 53% reduction in progression risk vs. High Risk (HR = 0.473, 95% CI: 0.197–1.134, *p* = 0.093; C-index = 0.688). Results are summarized in Table 3 and Figure 5 and Figure 6.

## 4. Discussion

In this transcriptomic and immune deconvolution analysis of 27 NSCLC patients treated with ICI, we identified a convergent immunosuppressive transcriptomic signature indicative of early progression. The concurrent activation of TGF-β signaling, WNT/β-catenin, and EMT programs, together with MYC/E2F-driven oncogenic proliferation, suggests that early progression is characterized by a reinforcing circuit of simultaneous immunosuppression and unrestrained tumor proliferative activity.

The dominant enrichment of TGF-β signaling (NES = 1.616, FDR = 0.0015) is biologically compelling. TGF-β promotes CD8^+^ T cell exclusion, Treg expansion, M2 macrophage polarization, and CAF activation [10,11]. The concurrent enrichment of WNT/β-catenin (NES = 1.660, FDR = 0.0051) provides an additional mechanistically distinct route to immune exclusion via suppression of Batf3^+^ dendritic cell recruitment [12,13]. EMT enrichment (NES = 1.430, FDR = 0.0009) reflects upregulation of PD-L1 and CAF activation [14,15]. MYC and E2F programs coordinate immunosuppression with enhanced proliferative capacity [16].

The 87-gene immunosuppressive risk score demonstrated a statistically significant association with outcome within this exploratory cohort (Spearman ρ = −0.516, *p* = 0.006). The C-index of 0.688 is comparable to or exceeds that reported for individual biomarkers such as PD-L1 IHC in unselected NSCLC populations. While the Kaplan–Meier and Cox results were nominally significant (*p* ≈ 0.09), the small sample size (*n* = 27) severely limits statistical power; the observed effect size (HR = 2.12) provides a rationale for hypothesis-driven validation in larger, adequately powered cohorts, though this should be interpreted with caution given the limited sample size and absence of external validation [24].

Several limitations of this study warrant careful consideration. First, the small sample size (*n* = 27; EP *n* = 17, CB *n* = 10) is a fundamental constraint that increases the risk of false-positive findings across all analyses and renders Cox regression estimates highly unstable, as evidenced by the wide confidence intervals. Second, the 87-gene immunosuppressive risk score was both derived and evaluated within the same dataset (GSE135222), constituting an internal derivation without external validation; this substantially limits the generalizability of the findings, and independent validation in a separate ICI-treated NSCLC cohort is necessary. Third, this study lacks detailed annotation of NSCLC histological subtype, treatment line, PD-L1 expression status, STK11/KEAP1 mutation status, and smoking history. The cross-sectional design precludes longitudinal analysis. Bulk RNA-seq deconvolution by EPIC cannot resolve intra-compartment heterogeneity that single-cell approaches would capture.

Future directions should include: (1) external validation of the 87-gene risk score in independent NSCLC ICI cohorts; (2) integration of genomic data (TMB, STK11/KEAP1 status); (3) single-cell RNA-seq studies; and (4) prospective biomarker studies in trials of combination regimens targeting TGF-β or WNT/β-catenin alongside ICI [24].

## 5. Conclusions

In NSCLC patients receiving ICI therapy, early disease progression is characterized by simultaneous transcriptomic activation of TGF-β/WNT-mediated immunosuppressive programs, EMT-driven stromal remodeling, and MYC/E2F oncogenic signaling—suggesting an actively sustained multi-layered suppressive state rather than a passive lack of immune activation. An 87-gene immunosuppressive risk score derived from these pathways demonstrates a statistically significant negative correlation with PFS (Spearman ρ = −0.516, *p* = 0.006) within this exploratory cohort; however, given the small sample size and lack of external validation, these findings are hypothesis-generating and require independent prospective validation in adequately powered NSCLC ICI cohorts before any predictive biomarker claims can be substantiated. These findings advocate for rational design of combination therapeutic strategies concurrently targeting TGF-β signaling and WNT/β-catenin in conjunction with ICI.

## Figures and Tables

**Table 1:** Top Hallmark pathways enriched in the Early Progression group by GSEA (FDR < 0.05).

Pathway	NES	FDR *p*-Value	Biological Theme
WNT/β-catenin Signaling	1.660	0.0051	Immunosuppressive
TGF-β Signaling	1.616	0.0015	Immunosuppressive
Estrogen Response Early	1.557	<0.0001	Hormonal Signaling
MYC Targets V2	1.508	0.0093	Oncogenic/Proliferative
MYC Targets V1	1.469	0.0003	Oncogenic/Proliferative
Epithelial–Mesenchymal Transition	1.430	0.0009	Immunosuppressive/Invasive
E2F Targets	1.412	0.0011	Cell Cycle/Oncogenic
G2M Checkpoint	1.321	0.0067	Cell Cycle/Oncogenic

**Table 2:** EPIC-based immune cell deconvolution results according to early progression and clinical benefit groups.

Cell Type	EP Median	CB Median	*p*-Value	FDR	Direction	Interpretation
B cells	0.0034	0.0029	0.902	0.980	→	NS
CAFs	0.0311	0.0155	0.170	0.398	↑ EP	NS trend
CD4^+^ T cells	0.0403	0.0426	0.980	0.980	→	NS
CD8^+^ T cells	0.0151	0.0205	0.127	0.398	↓ EP	NS trend
Endothelial cells	0.0291	0.0085	0.011	0.077	↑ EP	NS after FDR
Macrophages	0.0112	0.0205	0.334	0.584	↓ EP	NS
NK cells	0.0002	0.0008	0.786	0.980	↓ EP	NS

EP = Early Progression; CB = Clinical Benefit; NS = not significant. No cell type reached FDR-adjusted statistical significance. Endothelial cells (*p* = 0.011, FDR = 0.077) did not retain significance after Benjamini–Hochberg correction and should not be interpreted as a confirmed finding. All differences should be treated as exploratory trends only.

**Table 3:** Immunosuppressive risk score—survival analysis summary. All analyses exploratory; wide confidence intervals reflect limited sample size (*n* = 27).

Analysis	Result	*p*-Value
Spearman Correlation (score vs. PFS)	ρ = −0.516	0.006
Log-rank test (High vs. Low Risk)	Separation favoring Low Risk	0.088
Cox HR (Low vs. High Risk)	HR = 0.473 (95% CI: 0.197–1.134)	0.093
Concordance Index	0.688 (SE = 0.054)	—

## Data Availability

The datasets analyzed in this study are publicly available in the Gene Expression Omnibus (GEO) repository under accession number GSE135222. Further processed data and analysis outputs are available from the corresponding author upon reasonable request.
